# Randomized Trial of Mindfulness-Based Stress Reduction in Cardiac Patients Eligible for Cardiac Rehabilitation

**DOI:** 10.1038/s41598-019-54932-2

**Published:** 2019-12-05

**Authors:** Prabhjot S. Nijjar, John E. Connett, Ruth Lindquist, Roland Brown, Marsha Burt, Aaron Pergolski, Alexandra Wolfe, Priya Balaji, Nitya Chandiramani, Xiaohui Yu, Mary Jo Kreitzer, Susan A. Everson-Rose

**Affiliations:** 1 Cardiovascular Division, Department of Medicine, University of Minnesota Medical School, Minneapolis, Minnesota USA; 2 Biostatistics, Epidemiology and Research Design (BERD), University of Minnesota, Minneapolis, Minnesota USA; 3 Clinical and Translational Science Institute, University of Minnesota, Minneapolis, Minnesota USA; 4 School of Nursing, University of Minnesota, Minneapolis, Minnesota USA; 5 Cardiac Rehabilitation, Fairview Health Services, Minneapolis, Minnesota USA; 6 Program in Health Disparities Research, Department of Medicine, University of Minnesota Medical School, Minneapolis, Minnesota USA; 7 Earl E Bakken Center for Spirituality & Healing, University of Minnesota, Minneapolis, Minnesota USA

**Keywords:** Health care, Cardiology

## Abstract

Currently, exercise-based cardiac rehabilitation (CR) is the only recommended secondary prevention strategy for cardiac patients that attempts to tackle stress and psychosocial wellbeing, but it is under-utilized and lacks a comprehensive curriculum for this purpose; hence there is a critical gap to address psychosocial needs of cardiac patients after an event. Mindfulness-based stress reduction (MBSR) has shown benefits in the general population but its role in cardiac patients is not clear. We conducted a pilot randomized controlled trial (RCT) of MBSR in CR-eligible cardiac patients during their initial year of recovery. Patients were allocated 2:1 (intervention:control) to an 8-week MBSR group intervention or usual care. Standard measures of depression, anxiety, perceived stress, health related quality of life (HRQOL), blood pressure, biomarkers (lipids, HbA1c, CRP) and 24-hour Holter monitoring were obtained at baseline, 3- and 9-months post-randomization. Sub-group analyses were performed for participants with at least mild depression (PHQ-9 ≥ 5). 47 patients [mean age 58.6 years; 38% female; 77% white] were enrolled in 2 cohorts. 87% of MBSR patients completed the intervention; study retention was >95% at each follow-up visit. At 3 months, compared to controls, MBSR patients showed improvements in depression [p = 0.01] and anxiety [p = 0.04] with a similar trend in HRQOL [p = 0.06]. The MBSR group showed greater improvement or less worsening of most CV risk factors, with an attenuation of treatment effects at 9 months. Participants with at PHQ-9 scores ≥5 at baseline showed greater improvement in psychosocial and CV outcomes, that persisted at 9 months. MBSR is a safe and well received secondary prevention strategy. This pilot RCT provides preliminary evidence of MBSR’s potential to improve short term psychosocial well-being in cardiac patients during their first year of recovery.

## Introduction

Up to 40% of cardiac patients experience significant levels of depression, anxiety, and stress following a myocardial infarction (MI) or other serious cardiac events or procedures^1–4^. Such negative emotions contribute to poorer mental health and create challenges and barriers for patients in making and maintaining important behavioral and lifestyle modifications. This may increase health care costs and exacerbate risk for recurrent events, mortality and other adverse outcomes^5–7^. Although the American Heart Association (AHA) recognizes depression as a risk factor for poor prognosis and outcomes in cardiac patients^8^, effective treatments remain elusive. A recent meta-analysis showed little difference in effectiveness of pharmacologic treatment and individual psychotherapies on depression and anxiety disorders in the general population^9^. Moreover, in cardiac patients, traditional therapies have had limited effectiveness and have not impacted medical outcomes^10,11^. Pharmacologic treatments for depression and anxiety can have both cardiac and non-cardiac side effects, particularly in older populations^12,13^. Thus, there is a need to identify non-traditional treatment approaches that may alleviate depression and anxiety in cardiac patients.

For cardiac patients, the guideline-recommended standard of care following MI, heart failure, cardiac surgery, and other major cardiovascular events and procedures is individualized, exercise-based cardiac rehabilitation (CR)^14^. Though CR is an important secondary prevention strategy for millions of cardiac patients and has well-documented health benefits^15^, it lacks a comprehensive curriculum to address stress and psychosocial wellbeing. Moreover, CR is consistently underutilized, with poor enrollment, attendance and completion rates^16,17^, suggesting that additional secondary prevention strategies are needed.

One non-traditional treatment that is promising for cardiac patients but which has not been rigorously evaluated with this patient population is Mindfulness-Based Stress Reduction (MBSR). MBSR combines breathing practices, mindfulness meditation, and gentle yoga to cope with chronic illnesses and pain^18^. Prior studies in the general population have reported that MBSR can reduce symptoms of depression, anxiety and stress^19–21^. MBSR or components of MBSR may improve blood pressure^22^, immune function^23^, and autonomic nervous system activation^24^ although these associations have not been extensively studied. Additionally, MBSR potentially can enhance heart rate variability (HRV)^25,26^, an important indicator of sympatho-vagal balance and flexible emotional regulation that typically is reduced in cardiac patients^27–29^. MBSR is a widely used mind-body practice, but its efficacy in improving emotional regulation in cardiac patients within the first year of recovery is unknown. A 2017 scientific statement from the AHA systematically reviewed available data on potential benefits of meditative practices, including MBSR, on cardiovascular risk reduction^30^. The authors concluded that meditation-based practices are promising low-cost, low-risk interventions that should be considered as adjunct to guideline-recommended cardiovascular (CV) risk reduction approaches, but called for further research with methodologically rigorous study designs.

The present report is from the Mindful Heart Study (ClinicalTrials.gov #NCT02722213, registered on 29/03/2016), a pilot randomized controlled trial (RCT) of an 8-week MBSR group intervention versus usual care in patients currently eligible for guideline-recommended cardiac rehabilitation (hereon referred as CR-eligible). The primary goals of the study were to (1) establish feasibility of recruitment and retention strategies with this patient population, and (2) estimate treatment effects and variability of MBSR on levels of depression, anxiety, stress, health-related quality of life, and CV risk factors and biomarkers.

## Results

### Participant characteristics

For the final cohort of 47 patients, mean age was 58.6 years, 38.3% were women, and 76.6% were non-Hispanic white. On average, participants had experienced their qualifying cardiac event 22.7 weeks prior to study enrollment. At baseline, 22 (46.8%) self-reported that they were currently enrolled in phase 2 exercise-based CR. The most common indication for CR was CAD (78.7%) followed by surgical valve replacement (10.6%) and CHF (6.7%). The cohort was on good guideline directed medical therapy, with well controlled BP (mean BP: 115/71 mmHg) and lipids (mean LDL-C: 61 mg/dl). There was a high prevalence of depression (21.3% with PHQ-9 ≥ 10 indicating moderate depression) and anxiety (14.8% with GAD-7 ≥ 10 indicating moderate anxiety). Detailed participant characteristics are shown in Table 1.

**Table 1:** Participant Characteristics Overall and By Treatment or Control Group Assignment.

*Demographics*	Overall		Control		MBSR	
	(N = 47)		(N = 16)		(N = 31)	
	Mean (SD)	N (%)	Mean (SD)	N (%)	Mean (SD)	N (%)
Age, years	58.6 (10.8)		60.7 (9.32)		57.5 (11.5)	
Men		29 (61.7%)		12 (75.0%)		17 (54.8%)
**Race**						
Non-Hispanic White		37 (78.7%)		13 (81.2%)		24 (77.4%)
Black		5 (10.6%)		1 (6.2%)		4 (12.9%)
Hispanic		1 (2.1%)		1 (6.2%)		0 (0.0%)
Other		5 (10.6%)		0 (0.0%)		5 (16.1%)
**Education, highest level attained**						
High school diploma or equivalent		5 (10.6%)		1 (6.2%)		4 (12.9%)
Some college		13 (27.7%)		6 (37.5%)		7 (22.6%)
College degree or higher		29 (61.7%)		9 (56.2%)		20 (64.5%)
**Smoking status**						
Never		28 (59.6%)		10 (62.5%)		18 (58.1%)
Ever		19 (40.4%)		6 (37.5%)		13 (41.2%)
***Medical History***						
Indication for Cardiac Rehab						
CAD (stable angina, MI, PCI, CABG)		37 (78.7%)		13 (81.2%)		24 (77.4%)
Heart failure		3 (6.4%)		1 (6.2%)		2 (6.5%)
Cardiac surgery (valve replacement)		5 (10.6%)		1 (6.2%)		4 (12.9%)
Other		2 (4.3%)		1 (6.2%)		1 (3.2%)
History of diabetes		10 (21.3%)		3 (18.8%)		7 (22.6%)
History of hypertension		33 (70.2%)		12 (75.0%)		21 (67.7%)
***Medication Usage***						
Beta-blockers		37 (78.7%)		15 (93.8%)		22 (71.0%)
Aspirin		42 (89.4%)		15 (93.8%)		27 (87.1%)
Statin		39 (83.0%)		14 (87.5%)		25 (80.6%)
ACE-I/ARB		26 (55.3%)		11 (68.8%)		15 (48.4%)
BMI, kg/m^2^	29.5 (6.63)		29.3 (6.4)		29.6 (6.85)	
Systolic blood pressure, mmHg	115 (13.3)		109 (9.43)		118 (14.1)	
Diastolic blood pressure, mmHg	70.7 (9.77)		68.2 (7.65)		72.0 (10.6)	
Resting heart rate	71.2 (10.9)		72.2 (11.8)		70.8 (10.7)	
HDL cholesterol, mg/dL	48.1 (15.1)		46.1 (12.1)		49.1 (16.4)	
LDL cholesterol, mg/dL	61.0 (26.1)		58.3 (32.3)		62.3 (23.2)	
Triglycerides, mg/dL	151 (92.0)		151 (85.8)		150 (96.3)	
Hemoglobin A1C, %	5.93 (1.03)		5.85 (0.57)		5.97 (1.22)	
CRP, mg/l	3.01 (5.22)		1.03 (1.34)		3.96 (6.09)	
Heart rate variability measured by 24-hour average RMSSD (n = 40)	81.7 (46.4)		66.4 (36.9)		88.2 (49.0)	
***Psychosocial Factors***						
Depression, PHQ-9	5.62 (5.32)		3.81 (3.47)		6.56 (5.9)	
Anxiety, GAD-7	5.35 (4.36)		3.93 (2.66)		6.03 (4.87)	
Perceived Stress Scale	14.8 (7.34)		12.7 (5.63)		15.8 (7.92)	
**Health Related Quality of Life No. mentally unhealthy days past month**						
Health Related Quality of Life	5.5 (7.63)		2.94 (4.2)		6.87 (8.7)	
Self-rated health (1 = excellent to 5 = poor)	2.76 (0.92)		2.5 (0.73)		2.9 (0.99)	

### Sub-group of participants with PHQ-9 ≥ 5

Among the total cohort of 47, 20 participants had PHQ-9 ≥ 5 at baseline, indicating at least mild depression. This sub-group of participants with PHQ-9 ≥ 5 also had significantly higher baseline levels of anxiety (P < 0.001), stress (P = 0.014), and poorer HRQOL (P = 0.004) compared to the sub-group with PHQ-9 < 5 (Supplementary Table 1). Similarly, participants with PHQ-9 ≥ 5 had higher baseline levels of SBP (117 vs 113), DBP (72.3 vs 69.6), triglycerides (167 vs 138), HbA1c (6.3 vs 5.6), and hsCRP (3.47 vs 2.65).

### Retention and acceptability

Retention throughout the study exceeded 95%. One participant was deceased secondary to metastatic cancer prior to the first follow-up visit; among the 46 surviving participants, retention was 95.7% at the 3-month follow-up, and 97.8% at both the 6-month telephone follow-up and 9-month follow-up assessment.

Two MBSR groups were completed as part of the study. Acceptability and satisfaction with the intervention were strong. In response to the question “How important has MBSR been to you (scores ranged from 1, not at all important, to 5, very important), means (SD) were 4.5 (0.7). All respondents reported making lifestyle changes as a result of participating in the MBSR intervention. Additionally, average reported change in ability to handle stressful situations as a result of taking MBSR was 4.0 (SD, 0.6) (scores ranged from 1, great negative change, to 5, great positive change). From a total of 31 participants allocated to MBSR, 27 completed the intervention, with 96.3% (26 of 27) of those who completed the intervention missing ≤2 of the 9 MBSR sessions. There were no complications related to participation in MBSR.

### Psychosocial outcomes

Although this pilot RCT was designed as a feasibility study and was not powered to detect statistically significant group differences, we observed a positive benefit of the intervention on psychosocial outcomes. Relative to Controls, patients in the MBSR group experienced improved emotional regulation and psychosocial well-being at the 3-month follow-up, as measured by self-report of depressive symptoms, anxiety, perceived stress, and two indicators of HRQOL: the number of mentally unhealthy days experienced in the past month and self-rated health (Table 2). The change was statistically significant for depression (P = 0.01) and anxiety (P = 0.04), with a non-significant trend for perceived stress and HRQOL. While the average 3-month change in PHQ9 scores in the MBSR group was −2.3 points, nearly one-third (32.3%) of the MBSR group subjects had a 4-point or better improvement on the PHQ9 at the 3-month follow-up assessment, compared to just 6.2% of Control subjects. Similarly, 48.4% of MBSR group subjects showed a 4-point or better improvement on the GAD7, whereas only 12.5% of Control subjects had this level of improvement on the GAD7 at 3 months post-randomization (data not shown). There was an attenuation of treatment effect at 9 months, with none of the variables showing any significant change from baseline compared to control group (Table 2).

**Table 2:** Change in Psychosocial Outcomes: Mindful Heart Study RCT.

PHQ-9 (Depression)	*Change at 3 months*		*Change at 9 months*	
	Mean (SD)	Adjusted difference (95% CI)	Mean (SD)	Adjusted difference (95% CI)
MBSR	−2.3 (3.48)	−2.69 (−4.70, −0.68)**	−2.11 (3.76)	−1.18 (−3.67, 1.31)
Control	0.33 (2.26)		−0.93 (4.23)	
**GAD-7 (Anxiety)**				
MBSR	−3.03 (4.33)	−2.75 (−5.37, −0.13)*	−2.23 (4.34)	−0.89 (−3.37, 1.59)
Control	−0.32 (2.88)		−1.4 (2.31)	
**Perceived Stress Scale**				
MBSR	−3.39 (6.3)	−2.48 (−6.65, 1.69)	−4.13 (7.77)	−1.33 (−5.72, 3.07)
Control	−1.0 (6.24)		−2.93 (4.08)	
**Mentally unhealthy days in the last month**				
MBSR	−3.76 (7.03)	−3.93 (−8.04, 0.17)^†^	−2.55 (8.65)	−2.98 (−7.78, 1.81)
Control	0.07 (4.91)		0.47 (4.09)	
**Self-rated health**				
MBSR	−0.14 (0.79)	−0.26 (−0.74, 0.22)	−0.28 (0.75)	−0.41 (−0.87, 0.06)^‡^
Control	0.13 (0.64)		0.13 (0.64)	

N = 47 (31 randomized to MBSR; 16 randomized to Control).

**p = 0.01; *p = 0.04; ^†^p = 0.06; ^‡^p = 0.084.

Each measure is scored such that a higher score indicates more depressive symptoms, anxiety, etc. Values shown are means (SD) for 3-month and 9-month changes in the measures listed, and differences in change scores (95% CI) between the MBSR and control groups, adjusted for the randomization strata (i.e., concurrent enrollment in exercise-based cardiac rehabilitation [yes/no] at baseline). All change scores are calculated by subtracting the baseline value from the follow-up value at 3-months and at 9-months for a given measure. Thus, a negative change score reflects an improvement in these indicators from baseline whereas a positive change score represents a worsening of symptoms from baseline.

### Cardiovascular outcomes

At 3-months post-randomization, we observed a trend toward improved SBP in the MBSR group compared to controls: 3-month SBP **∆** was −2.02 (SD, 9.11) mmHg for the MBSR group and +1.89 (SD, 7.79) mmHg for the control group (difference (95% CI) = −3.84 (−9.57, +1.90) (p = 0.18)). There was little differentiation between MBSR and Control groups on 3-month changes in DBP, total cholesterol, LDL or HDL cholesterol, triglycerides, BMI, HbA1c, hsCRP, or RMSSD (Table 3).

**Table 3:** Change in Cardiovascular Outcomes: Mindful Heart Study RCT.

SBP	*Change at 3 months*		*Change at 9 months*	
	Mean (SD)	Adjusted difference (95% CI)	Mean (SD)	Adjusted difference (95% CI)
MBSR	−2.02 (9.11)	−3.84 (−9.57, 1.90)	5.12 (13.7)	−1.35 (−9.82, 7.12)
Control	1.89 (7.79)		6.47 (12.3)	
**DBP**				
MBSR	0.12 (6.34)	1.08 (−3.23, 5.38)	2.97 (7.82)	−1.13 (−6.43, 4.17)
Control	−0.89 (7.01)		4.1 (9.29)	
**Heart Rate**				
MBSR	1.79 (4.94)	1.14 (−2.89, 5.17)	−0.03 (8.65)	−2.50 (−8.08, 3.08)
Control	0.67 (7.15)		2.47 (8.76)	
**Body Mass Index**				
MBSR	0.68 (2.02)	0.14 (−1.10, 1.38)	0.98 (2.16)	−0.32 (−1.79, 1.16)
Control	0.55 (1.49)		1.29 (2.54)	
**Total Cholesterol**				
MBSR	9.2 (38.2)	1.54 (−21.35, 24.43)	−2.03 (22.4)	−7.72 (−23.11, 7.66)
Control	7.69 (19.7)		5.43 (25.9)	
**LDL Cholesterol**				
MBSR	12.5 (32.7)	5.44 (−15.07, 25.94)	0.24 (22.5)	−1.83 (−17.05, 13.39)
Control	7.08 (16.7)		2.08 (21.6)	
**HDL Cholesterol**				
MBSR	1.13 (5.59)	−1.87 (−5.44, 1.71)	1.97 (8.03)	−1.25 (−6.30, 3.81)
Control	3.0 (4.38)		3.29 (6.96)	
**Triglycerides**				
MBSR	−25.0 (77.7)	−5.59 (−53.20, 42.01)	−17.4 (72.7)	−13.99 (−57.54, 29.57)
Conntrol	−19.3 (52.7)		−5.14 (67.6)	
**HbA1c**				
MBSR	0.07 (0.56)	0.06 (−0.27, 0.39)	0.09 (0.78)	−0.16 (−0.59, 0.27)
Control	0.01 (0.29)		0.26 (0.47)	
**hsCRP**				
MBSR	0.15 (6.77)	−0.09 (−3.93, 3.75)	−0.58 (6.07)	−2.11 (−5.70, 1.48)
Control	0.25 (0.69)		1.49 (3.75)	
*RMSSD				
MBSR	5.52 (30.2)	6.51 (−14.33, 27.34)	−2.11 (26.9)	9.59 (−7.50, 26.69)
Control	−2.18 (18.5)		−12.8 (19.1)	

N = 47 (31randomized to MBSR; 16 randomized to Control).

*RMSSD = Root Mean Square of Successive Differences; a measure of heart rate variability (HRV) that represents the square root of the mean squared differences of successive R-R intervals obtained from portable 24-hour heart rate monitors worn at baseline and at the 3-month and 9-month follow-up visits. Due to equipment failure, data recordings were only available on 40 participants; with extreme outliers removed (n = 7), data shown are from N = 33 (21 in MBSR group and 11 in Control).

Values shown are means (SD) for 3-month and 9-month changes in the cardiovascular measures listed, and differences in change scores (95% CI) between the MBSR and Control groups, adjusted for the randomization strata (i.e., concurrent enrollment in exercise-based cardiac rehabilitation [yes/no] at baseline). All change scores are calculated by subtracting the baseline value from the follow-up value at 3-months and at 9-months for a given measure. With the exception of HDL cholesterol and RMSSD, higher values on the cardiovascular factors listed are associated with greater risk; in contrast, lower levels of HDL and lower HRV are associated with greater cardiovascular risk.

By the 9-month follow-up, the difference between groups for SBP was smaller (difference (95% CI) = −1.35 (−9.82, +7.12)) but still favored the MBSR group. The MBSR group showed either greater improvement or less worsening of other CV risk factors (with the exception of HDL) as shown in Table 3, although none of the differences was statistically significant.

### Psychosocial outcomes for participants with PHQ-9 ≥ 5

For the sub-group of 20 participants with PHQ-9 ≥ 5, those in the MBSR group (n = 15) showed consistently larger improvements in PHQ9, GAD7, PSS, and HRQOL at 3 months compared to the Control group (n = 5) (Table 4). Due to small numbers, none of these differences was statistically significant, but the observed trends are of interest as the magnitude of improvement seen in this sub-group was greater than for the entire cohort, particularly for the measures of anxiety, stress and HRQOL. As with the entire cohort, there was an attenuation of treatment effect at 9 months.

**Table 4:** Change in Psychosocial Outcomes in Participants with Baseline PHQ-9 > 5 (N = 20).

PHQ-9 (Depression)	*Change at 3 months*		*Change at 9 months*	
	Mean (SD)	Adjusted difference (95% CI)	Mean (SD)	Adjusted difference (95% CI)
MBSR	−3.93 (4.1)	−2.71 (−7.42, 1.99)	−3.27 (5.02)	0.73 (−4.85, 6.31)
Control	−1.5 (2.89)		−4.0 (5.05)	
**GAD-7 (Anxiety)**				
MBSR	−3.93 (5.65)	−3.61 (−9.83, 2.62)	−2.6 (5.91)	−1.23 (−6.94, 4.48)
Control	−0.75 (2.36)		−1.37 (3.44)	
**Perceived Stress Scale**				
MBSR	−5.73 (6.12)	−5.16 (−12.26, 1.94)	−4.53 (9.46)	−2.53 (−11.77, 6.71)
Control	−0.75 (3.59)		−2.0 (5.34)	
**Mentally unhealthy days in the last month**				
MBSR	−6.2 (9.04)	−8.24 (−17.90, 1.42)^‡^	−3.93 (11.9)	−5.93 (−17.78, 5.92)
Control	1.0 (5.72)		2.0 (5.66)	
**Self-rated health**				
MBSR	−0.33 (0.82)	−0.33 (−1.35, 0.68)	−0.33 (0.9)	−0.13 (−1.13, 0.86)
Control	0.0 (0.82)		−0.2 (0.84)	

The subgroup with baseline PHQ-9 scores of 5 or greater included 20 participants, 15 of whom had been randomized to the MBSR intervention group and 5 of whom had been randomized to the Control group.

^‡^p = 0.089

Each measure is scored such that a higher score indicates more depressive symptoms, anxiety, etc. Values shown are means (SD) for 3-month and 9-month changes in the measures listed, and differences in change scores (95% CI) between the MBSR and control groups, adjusted for the randomization strata (i.e., concurrent enrollment in exercise-based cardiac rehabilitation [yes/no] at baseline). All change scores are calculated by subtracting the baseline value from the follow-up value at 3-months and at 9-months for a given measure. Thus, a negative change score reflects an improvement in these indicators from baseline whereas a positive change score represents a worsening of symptoms from baseline.

### Cardiovascular outcomes for participants with PHQ-9 ≥ 5

Within the sub-group of participants with PHQ-9 ≥ 5, the MBSR group had improvement or less worsening of the following compared to Controls at 3 months (Table 5): total cholesterol, LDL-C, triglycerides, HbA1c, hsCRP, SBP, and RMSSD. These improvements persisted at 9 months, with the magnitude of change even greater for total cholesterol, LDL-C, HbA1c, hsCRP, SBP and RMSSD (Table 5).

**Table 5:** Change in Cardiovascular Outcomes in Participants with Baseline PHQ-9 > 5 (N = 20).

Systolic Blood Pressure	*Change at 3 months*		*Change at 9 months*	
	Mean (SD)	Adjusted difference (95% CI)	Mean (SD)	Adjusted difference (95% CI)
MBSR	−2.17 (9.83)	−5.93 (−17.51, 5.66)	8.0 (13.5)	−7.20 (−21.46, 7.06)
Control	3.38 (7.33)		15.2 (10.9)	
**Diastolic Blood Pressure**				
MBSR	−0.5 (7.66)	−0.36 (−9.38, 8.66)	4.97 (8.47)	−3.73 (−12.40, 4.93)
Control	−0.62 (6.42)		8.7 (6.78)	
**Heart Rate**				
MBSR	2.62 (4.66)	−0.48 (−6.86, 5.91)	−1.0 (10.2)	−6.00 (−16.96, 4.96)
Control	3.0 (6.48)		5.0 (8.6)	
**Body Mass Index**				
MBSR	0.97 (2.71)	0.92 (−2.28, 4.12)	1.4 (2.84)	0.43 (−2.69, 3.55)
Control	0.06 (1.89)		0.97 (2.57)	
**Total Cholesterol**				
MBSR	1.8 (33.1)	−21.83 (−59.09, 15.44)	−7.4 (23.8)	−26.60 (−50.74, −2.46)**
Control	22.0 (13.6)		19.2 (12.3)	
**LDL Cholesterol**				
MBSR	7.5 (28.1)	−13.90 (−50.97, 23.18)	−5.93 (21.6)	−21.95 (−46.79, 2.89)‡
Control	19.7 (26.4)		17.0 (18.7)	
**HDL Cholesterol**				
MBSR	1.6 (3.96)	0.21 (−4.34, 4.75)	3.0 (8.16)	2.00 (−7.30, 11.30)
Control	1.25 (2.06)		1.0 (9.0)	
**Triglycerides**				
MBSR	−42.2 (78.2)	−35.38 (−129.38, 58.62)	−16.7 (75.3)	−13.99 (−57.54, 29.57)
Control	−3.25 (69.6)		6.4 (53.8)	
**HbA1c**				
MBSR	0.0 (0.54)	−0.10 (−0.69, 0.49)	0.01 (0.86)	−0.49 (−1.26, 0.28)
Control	0.07 (0.1)		0.54 (0.63)	
**hsCRP**				
MBSR	−0.09 (5.75)	−0.31 (−6.20, 5.58)	−1.61 (5.48)	−5.17 (−11.31, 0.97)†
Control	0.88 (0.91)		3.56 (6.01)	
*RMSSD				
MBSR	7.11 (37.0)	−8.50 (−65.32, 48.32)	4.09 (27.0)	10.29 (−20.88, 41.47)
Control	5.0 (35.0)		−10.8 (21.4)	

The subgroup with baseline PHQ-9 scores of 5 or greater included 20 participants, 15 of whom had been randomized to the MBSR intervention group and 5 of whom had been randomized to the Control group.

**p = 0.033; ^‡^p = 0.079; ^†^p = 0.093.

*RMSSD = Root Mean Square of Successive Differences; a measure of heart rate variability (HRV) that represents the square root of the mean squared differences of successive R-R intervals obtained from portable 24-hour heart rate monitors worn at baseline and at the 3-month and 9-month follow-up visits. Due to equipment failure, data recordings were only available on 40 participants; with extreme outliers removed (n = 7), data shown are from N = 33 (21 in MBSR group and 11 in Control).

Values shown are means (SD) for 3-month and 9-month changes in the cardiovascular measures listed, and differences in change scores (95% CI) between the MBSR and control groups, adjusted for the randomization strata (i.e., concurrent enrollment in exercise-based cardiac rehabilitation [yes/no] at baseline). All change scores are calculated by subtracting the baseline value from the follow-up value at 3-months and at 9-months for a given measure. With the exception of HDL cholesterol and RMSSD, higher values on the cardiovascular factors listed are associated with greater risk; in contrast, lower levels of HDL and lower HRV are associated with greater cardiovascular risk.

### Associations between psychosocial and cardiovascular outcomes

**Figure 1:**
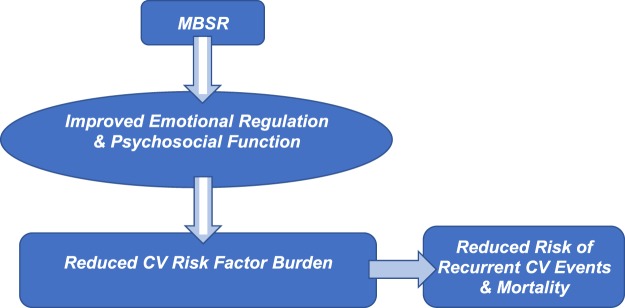
Conceptual Model of MBSR in Cardiac Patients.

In keeping with our conceptual model of MBSR wherein psychosocial improvement leads to CV risk factor improvement over time (Fig. 1), we assesed whether 3-month psychosocial outcomes were associated with 9-month cardiovascular outcomes. For the entire sample, 3-month PHQ9 scores were meaningfully associated with 9-month values for SBP (*r = *0.44, *p = *0.003), BMI (*r = *0.32, *p = *0.036), HbA1c (*r = *0.34, *p = *0.025), and triglycerides (*r = *0.26, *p = *0.10) in age and sex-adjusted analyses. GAD7 scores at 3 months were similarly associated with these CV risk factors at 9 months (adjusted *r*s = 0.33 (SBP), 0.24 (BMI), 0.31 (HbA1c) and 0.17 (triglycerides)).

## Discussion

In this pilot RCT designed to establish feasiblity and safety and estimate potential treatment effects of MBSR in cardiac patients, we found MBSR was safe and well accepted with high completion rate, in a cohort of 47 patients with a recent cardiac event who were eligible for CR. Compared to usual care, participation in MBSR led to statistically significant improvements in depression and anxiety at 3-months, an effect that was attenuated at 9 months. There was a trend to improvement or less worsening of cardiometabolic outcomes at 3-months and 9-months, though not statistically significant.

Despite the well-documented health benefits of CR, it continues to be hindered by poor attendance and completion rates^16,17^. Due to this, the research and clinical community may have concerns about enrolling CR eligible patients in another intensive lifestyle program such as MBSR. We were able to demonstrate excellent recruitment and retention in this patient population. Our study design to allow participation in the trial independent of whether patients enrolled in CR likely helped with recruitment. Even though all CR-eligible patients could participate regardless of qualifying condition, most of the participants had CAD rather than CHF.

A large percentage of cardiac patients experience significant symptoms of depression and anxiety^1–4^, which are associated with excess morbidity and mortality^5–8^. Even mild symptoms of depression after a cardiac event are associated with increased risk of cardiac mortality over 5 years^31^. In one study of hospitalized cardiac patients, each 1-point higher PHQ9 score was related to a significant 9% increased odds of 6-month cardiac readmission^32^. With respect to depression outcomes, a 3 to 5 point improvement in PHQ9 scores is considered clinically meaningful^33^. Nearly one-third and one-half of the MBSR group subjects had a 4-point or better improvement on the PHQ9 and GAD7, respectively, at the 3-month follow-up assessment.

We found a significant attenuation of treatment effect on psychosocial outcomes at 9 months, with none of the variables showing any significant change from baseline compared to control group. After completing the 8-week MBSR course, participants are encouraged to continue their Mindfulness practice and the various exercises. However, there is no formal curriculum or follow up once the course is complete. These findings suggest a dose effect of MBSR whereas the beneficial effects of the course start to wane over time, and a re-dose may be necessary to enjoy continued benefits of the MBSR training. Future studies should address this issue, and test the optimal strategy for re-dosing (repeat full course vs a shorter refresher).

A small trend for improvement was observed for SBP at 3-months follow-up in the MBSR group. There was little differentiation between MBSR and Control groups on other CV risk factors at 3 months. However, by 9 months, the MBSR group showed either greater improvement or less worsening of most CV risk factors, though not statistically significant. The overall worsening of many CV risk factors compared to baseline in the entire cohort reflects the ongoing need for optimizing secondary prevention strategies in these patients.

We observed that 3-month PHQ9 and GAD7 scores were meaningfully associated with 9-month values for SBP, BMI, HbA1c, and triglycerides. This pattern of associations is consistent with the extant literature showing that higher levels of depression and anxiety are related to greater cardiometabolic risk over time in this patient population^34^, and consistent with our conceptual model (Fig. 1).

When stratified by the presence of at least mild depression (PHQ-9 ≥ 5), a sub-group of participants was identified that had worse mental well-being and CV risk factors at baseline. This sub-group showed improvements in psychosocial and CV outcomes with MBSR, with magnitude of change greater than observed for the entire cohort. In contrast to the entire cohort, the improvement in CV outcomes in this sub-group persisted at 9-months.

Several large RCTs have shown no benefit of stress reduction training in reducing stress and improving clinical outcomes^35–37^. All these studies used varied stress reduction therapies that were used as the sole intervention, and not as part of a multi-faceted approach like CR. Blumenthal *et al*. randomized 151 patients with CAD to CR or CR and stress management training (SMT)^38^. The SMT intervention was based upon a cognitive-behavioral therapy model and delivered in 12 weekly 1.5- hour sessions. In the CR + SMT group compared to CR only group, there were significant improvements in patient reported stress and adverse clinical events. The study by Blumenthal *et al*. was different from the other RCTs in that it was effective at reducing stress, which in turn likely led to improved clinical outcomes. The SMT intervention tested in the trial is not widely disseminated or practiced, whereas the MBSR program offers the advantage of being standardized and readily available at many sites nationwide. The standardized nature of MBSR make it ideal to be tested rigorously in diverse patient populations, and its large national footprint makes it ideally placed to be rolled out with good patient access if found to be successful at improving outcomes.

## Limitations

This was a small single center pilot study, designed primarily to establish feasbility of recruitment and retention strategies for this population of cardiac paitents within their first year of recovery. Thus, the study was not powered to detect statistically significant differences in outcomes. Even though all CR eligible patients were included, most patients had CAD and hence the findings may not be generizable to all cardiac patients. The improvement in psychosocial outcomes was significantly attenuated at 9 months, which could reflect regression to the mean. However, given the continued improvement, we think this more likely reflects waning of the treatment effect of MBSR. There was no significant change in the tested CV outcomes, though as stated this likely reflects the small sample size. Due to the nature of the intervention, patients were not blinded and therefore a placebo effect cannot be excluded. Research volunteers may have been especially motivated to participate in the program, which may limit the generalizability of our findings.

## Conclusions

MBSR is a safe and well received secondary prevention strategy for cardiac patients. This pilot RCT provides preliminary evidence of MBSR’s potential to improve short term psychosocial well-being in cardiac patients during their first year of recovery. The improvement was attenuated at 9 months, and future studies should consider providing a booster dose of MBSR for a persistent effect. Stratification by the presence of at least mild depression (PHQ-9 ≥ 5) identified a sub-group of participants with worse mental well-being and CV risk factors, and this sub-group showed greater improvements in psychosocial and CV outcomes with MBSR. Further research is needed to evaluate effects of MBSR on mental health and CV risk factors in a diverse patient population, with likely more impact in patients with at least mild depression.

## Methods

### Study population

Men and women age 21 and older with a qualifying cardiac event or procedure and referral to CR within the past 12 months, no prior experience with MBSR, and willing to participate in all assessments were eligible for the study. As CR is the guideline recommended standard of care for our target patient population, we included eligibility for CR as an inclusion criterion; however, because attendance at CR is variable and typically low among referred patients, CR enrollment was not a requirement. Patients with pacemaker dependency or uncontrolled arrhythmias were excluded. Patients were recruited from a large university-affiliated hospital system at different points of contact with the care system, including via letters to eligible patients, on-site at the CR settings, and through advertising in cardiology clinic locations. The Institutional Review Board at the University of Minnesota approved the study and all patients provided written, informed consent. All methods were performed in accordance with the relevant guidelines and regulations.

### Procedures

**Figure 2:**
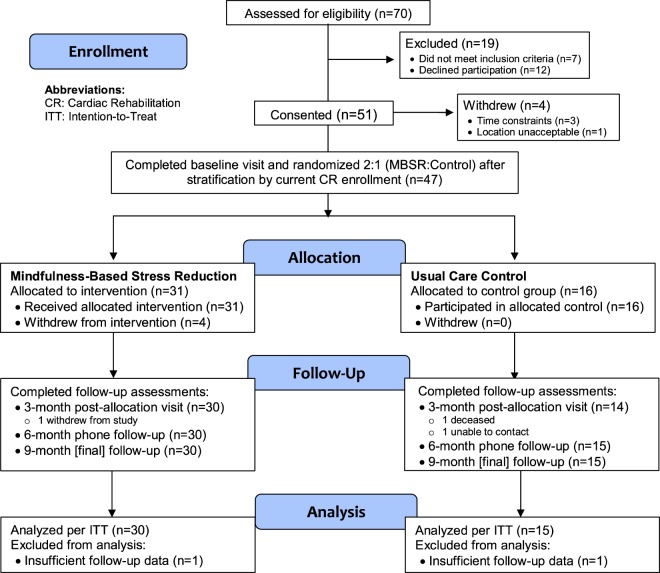
CONSORT Flow Diagram.

The study included a telephone screening to determine eligibility, three in-person assessments, and one telephone follow-up assessment. The study employed an IRB-approved e-consent process whereby eligible and interested participants were given a secured website link to the informed consent form where they could read and sign the consent form electronically. This could be completed remotely in advance of the in-person baseline visit, an option selected by 66.8% of enrollees. The in-person assessments included a baseline visit (pre-randomization), and one follow-up visit conducted at 3 months post-randomization, and a second follow-up visit 9 months post-randomization. The telephone follow-up occurred at the mid-point between the two in-person follow-up assessments. Participants were given monetary incentives for completion of each assessment. At the end of the baseline visit, participants were stratified based on self-reported current CR enrollment (yes/no), and then randomly allocated to an 8-week MBSR group intervention or usual care control group using a 2:1 (intervention:control) randomization scheme by the study coordinator, blinded to study investigators. We used separate randomization schedules for each stratum, with permuted blocks of small size to achieve approximate balance between treatment assignments at any point in the schedule. Randomization was implemented using a REDCap program, which permits web-based data entry and randomization assignments. All participants received standard printed materials on healthy lifestyles and stress management; control group participants also received a CD and workbook on MBSR and proven stress-management techniques after completion of their 9-month follow-up visit. The intervention lasted for 8 weeks and was initiated within the first 3 weeks after randomization for all participants allocated to MBSR. A total of 70 potential participants were screened, of whom 51 (81% of eligible) consented and 47 were enrolled and allocated either to the 8-week MBSR intervention (n = 31) or usual care control (n = 16). Participants were recruited in two cohorts and 2 separate MBSR intervention groups were completed. Recruitment was completed within 15 weeks between June-September 2016, and all follow-up was completed by August 2017. Figure 2 depicts the CONSORT diagram for the study.

### Study intervention

The study intervention was the 8-week Mindfulness Based Stress Reduction course that was developed by Jon Kabat-Zinn and colleagues at the University of Massachusetts in the early 1980s in an attempt to integrate mindfulness meditation into mainstream clinical medicine^18^. MBSR is a combination of mindfulness meditation, breathing practices, and gentle yoga. It consists of eight 2.5-hour weekly sessions and one 6.5-hour retreat. MBSR teaches techniques and practices to participants according to a standard protocol that has been described previously^18^. MBSR instructors undergo intensive training and earn certification, assuring that delivery of the MBSR intervention is relatively invariant wherever and by whomever it is taught. In this study, both of the MBSR intervention groups were led by the same trained instructor from the University of Minnesota Earl E. Bakken Center for Spirituality and Healing.

## Outcome Variables

### Psychosocial measures

Based on our conceptual model of how MBSR may improve outcomes, we hypothesized that MBSR improves psychosocial wellbeing and emotional regulation, which in turn improves cardiometabolic and autonomic functioning leading to improved clinical outcomes (Fig. 2). Depressive symptoms, anxiety, perceived stress, and health-related quality of life (HRQOL) were measured at the baseline, 3-month and 9-month study visits by well-validated, self-report questionnaires commonly used in both clinical and research settings. Depressive symptoms were assessed with the Patient Health Questionnaire 9-item depression scale (PHQ9)^39^; anxiety was assessed by the Generalized Anxiety Disorder 7-item scale (GAD7)^40^, and perceived stress was measured by the 10-item Perceived Stress Scale^41^, an indicator of overall stress burden in a person’s life. The Center for Disease Control’s HRQOL questions assessed self-rated health and mentally unhealthy days over the past month^42^.

### CV risk factors and biomarkers

Health history, medication usage, and self-reported CR participation status were assessed at the baseline visit, with updates at subsequent study visits. Heart rate variability was measured by portable 24-hour heart rate monitors (*Cardiokey, Cardionet, Malvern, PA*), and analyzed by a computerized algorithm as recommended by a task force of the European Society of Cardiology and the North American Society of Pacing and Electrophysiology (now the Heart Rhythm Society)^43^. Root mean square of successive differences (RMSSD) is the square root of the mean squared differences of successive R-R intervals. RMSSD is considered to predominantly reflect parasympathetic influences^43^. Heart rate, systolic blood pressure (SBP) and diastolic blood pressure (DBP) were measured at each study visit. Blood draws for assays of hsCRP, HbA1c, and lipid profiles occurred at the in-person visits, immediately after resting blood pressure was measured, and prior to other assessment tasks.

### Statistical analysis

Sample size calculations were not done due to the pilot nature of the study. Descriptive statistics were tabulated overall and by treatment group. These included the means and standard deviations for continuous variables and frequencies with percentages for categorical variables. Outcomes were 3-month and 9-month changes in psychosocial and CV risk measures. Unadjusted and adjusted differences in mean outcome measures between MBSR participants and controls were evaluated using linear regression and the t-distribution with corresponding model degrees of freedom for confidence intervals and P-values. Adjusted differences included a term for cardiac rehabilitation strata in the regression model. All analyses were conducted using R v3.4.3^44^.

## Supplementary information

Supplementary Table 1
