# Supplementary material for: Randomized Trial of Mindfulness-Based Stress Reduction in Cardiac Patients Eligible for Cardiac Rehabilitation
Source: Sci Rep. 2019 Dec 5;9:18415. doi: 10.1038/s41598-019-54932-2 (PMC6895078; doi:10.1038/s41598-019-54932-2)
Supplement: Supplementary file 1 — Supplementary Table 1 [file 41598_2019_54932_MOESM1_ESM.docx]

Supplementary material for:

**Randomized Trial of Mindfulness-Based Stress Reduction in Cardiac Patients Eligible for Cardiac Rehabilitation**

Prabhjot S. Nijjar, MD; John E. Connett, PhD; Ruth Lindquist, PhD, RN; Roland Brown; Marsha Burt; Aaron Pergolski; Alexandra Wolfe; Priya Balaji; Nitya Chandiramani; Xiaohui Yu; Mary Jo Kreitzer, PhD, RN; Susan A Everson-Rose, PhD, MPH

**Supplementary Table 1. Baseline Psychosocial and CV Risk Factors Stratified by Baseline PHQ-9 Scores**

| **Variables** | **Overall** | **PHQ-9 < 5 at baseline** | **PHQ-9 > 5 at baseline** | **Difference in**  **Means (95% CI)** | **T-test P** |
| --- | --- | --- | --- | --- | --- |
|  | (N=47)  Mean (SD) | (N=27)  Mean (SD) | (N=20)  Mean (SD) |  |  |
| Baseline PHQ9 | 5.62 (5.32) | 1.87 (1.47) | 10.7 (4.29) | -8.83 (-10.91, -6.76) | < 0.001 |
| Baseline GAD7 | 5.35 (4.36) | 3.15 (2.34) | 8.2 (4.76) | -5.05 (-7.43, -2.66) | < 0.001 |
| Baseline PSS | 14.8 (7.34) | 12.3 (5.63) | 17.9 (8.21) | -5.55 (-9.92, -1.19) | 0.014 |
| Mentally Unhealthy Days | 5.5 (7.63) | 1.69 (3.21) | 10.4 (8.88) | -8.76 (-13.07, -4.45) | < 0.001 |
| Self-Rated Health | 2.76 (0.92) | 2.42 (0.81) | 3.2 (0.89) | -0.78 (-1.29, -0.26) | 0.004 |
| Baseline BMI | 29.5 (6.63) | 29.1 (6.6) | 30.1 (6.78) | -1.05 (-5.05, 2.95) | 0.598 |
| Baseline SBP | 115 (13.3) | 113 (12.6) | 117 (14.4) | -3.26 (-11.43, 4.91) | 0.424 |
| Baseline DBP | 70.7 (9.77) | 69.6 (8.92) | 72.3 (10.8) | -2.75 (-8.77, 3.27) | 0.36 |
| Baseline HDL | 48.1 (15.1) | 47.0 (12.2) | 49.5 (18.4) | -2.55 (-12.27, 7.17) | 0.596 |
| Baseline LDL | 61.0 (26.1) | 63.5 (24.7) | 57.4 (28.3) | 6.11 (-10.65, 22.87) | 0.464 |
| Baseline Triglycerides | 151 (92.0) | 138 (64.2) | 167 (118.8) | -29.91 (-90.18, 30.36) | 0.318 |
| Baseline HbA1C | 5.93 (1.03) | 5.63 (0.58) | 6.33 (1.36) | -0.69 (-1.38, -0.01) | 0.048 |
| Baseline hsCRP | 3.01 (5.22) | 2.65 (4.88) | 3.47 (5.72) | -0.82 (-4.06, 2.42) | 0.611 |
